# Supplementary material for: Role and mechanism of NCAPD3 in promoting malignant behaviors in gastric cancer
Source: Front Pharmacol. 2024 Apr 22;15:1341039. doi: 10.3389/fphar.2024.1341039 (PMC11070777; doi:10.3389/fphar.2024.1341039)
Supplement: Supplementary file 11 [file DataSheet2.ZIP › GSEA/Transcipt factor/my_analysis.Gsea.1599461814734/RTAAACA_FREAC2_01.html]

Details for gene set RTAAACA\_FREAC2\_01[GSEA]

|  || Dataset | filtered\_dataset.sample\_info.cls#WT\_versus\_NCAPD3\_MUT |
| Phenotype | sample\_info.cls#WT\_versus\_NCAPD3\_MUT |
| Upregulated in class | NCAPD3\_MUT |
| GeneSet | RTAAACA\_FREAC2\_01 |
| Enrichment Score (ES) | -0.36399373 |
| Normalized Enrichment Score (NES) | -2.7146845 |
| Nominal p-value | 0.0 |
| FDR q-value | 0.0025574518 |
| FWER p-Value | 0.002 |
Table: GSEA Results Summary

  

Fig 1: Enrichment plot: RTAAACA\_FREAC2\_01      
 Profile of the Running ES Score & Positions of GeneSet Members on the Rank Ordered List

  

| SYMBOL | TITLE | RANK IN GENE LIST | RANK METRIC SCORE | RUNNING ES | CORE ENRICHMENT || 1 | 51134 | CEP83 | 19 | 1.038 | 0.0053 | No |
| 2 | 53354 | PANK1 | 39 | 0.949 | 0.0089 | No |
| 3 | 5087 | PBX1 | 54 | 0.908 | 0.0155 | No |
| 4 | 23338 | JADE2 | 71 | 0.855 | 0.0196 | No |
| 5 | 56110 | PCDHGA5 | 102 | 0.781 | 0.0117 | No |
| 6 | 4193 | MDM2 | 131 | 0.769 | 0.0051 | No |
| 7 | 6925 | TCF4 | 132 | 0.768 | 0.0196 | No |
| 8 | 7248 | TSC1 | 158 | 0.718 | 0.0143 | No |
| 9 | 23023 | TMCC1 | 181 | 0.695 | 0.0108 | No |
| 10 | 4281 | MID1 | 294 | 0.595 | -0.0626 | No |
| 11 | 8125 | ANP32A | 326 | 0.578 | -0.0751 | No |
| 12 | 1203 | CLN5 | 358 | 0.562 | -0.0879 | No |
| 13 | 9075 | CLDN2 | 420 | 0.522 | -0.1241 | No |
| 14 | 805 | CALM2 | 423 | 0.518 | -0.1158 | No |
| 15 | 57727 | NCOA5 | 425 | 0.517 | -0.1068 | No |
| 16 | 27097 | TAF5L | 461 | 0.498 | -0.1238 | No |
| 17 | 79047 | KCTD15 | 469 | 0.493 | -0.1198 | No |
| 18 | 3479 | IGF1 | 511 | 0.469 | -0.1419 | No |
| 19 | 1979 | EIF4EBP2 | 532 | 0.456 | -0.1484 | No |
| 20 | 55252 | ASXL2 | 547 | 0.448 | -0.1504 | No |
| 21 | 4784 | NFIX | 567 | 0.439 | -0.1565 | No |
| 22 | 84335 | AKT1S1 | 572 | 0.437 | -0.1513 | No |
| 23 | 23786 | BCL2L13 | 588 | 0.432 | -0.1544 | No |
| 24 | 26100 | WIPI2 | 601 | 0.423 | -0.1555 | No |
| 25 | 5147 | PDE6D | 624 | 0.410 | -0.1644 | No |
| 26 | 149076 | ZNF362 | 680 | 0.388 | -0.1986 | No |
| 27 | 23767 | FLRT3 | 781 | 0.333 | -0.2679 | No |
| 28 | 8904 | CPNE1 | 814 | 0.308 | -0.2862 | No |
| 29 | 667 | DST | 886 | -0.303 | -0.3342 | No |
| 30 | 9181 | ARHGEF2 | 902 | -0.326 | -0.3394 | No |
| 31 | 3164 | NR4A1 | 912 | -0.341 | -0.3397 | No |
| 32 | 80352 | RNF39 | 931 | -0.355 | -0.3466 | No |
| 33 | 3489 | IGFBP6 | 934 | -0.359 | -0.3413 | No |
| 34 | 54498 | SMOX | 965 | -0.384 | -0.3567 | Yes |
| 35 | 6239 | RREB1 | 974 | -0.391 | -0.3554 | Yes |
| 36 | 7067 | THRA | 976 | -0.392 | -0.3487 | Yes |
| 37 | 10724 | MGEA5 | 983 | -0.394 | -0.3458 | Yes |
| 38 | 7090 | TLE3 | 984 | -0.395 | -0.3383 | Yes |
| 39 | 687 | KLF9 | 996 | -0.402 | -0.3390 | Yes |
| 40 | 9586 | CREB5 | 1009 | -0.409 | -0.3403 | Yes |
| 41 | 130271 | PLEKHH2 | 1012 | -0.412 | -0.3340 | Yes |
| 42 | 53349 | ZFYVE1 | 1018 | -0.417 | -0.3299 | Yes |
| 43 | 638 | BIK | 1024 | -0.421 | -0.3257 | Yes |
| 44 | 23764 | MAFF | 1039 | -0.436 | -0.3281 | Yes |
| 45 | 80005 | DOCK5 | 1045 | -0.441 | -0.3235 | Yes |
| 46 | 27111 | SDCBP2 | 1070 | -0.457 | -0.3330 | Yes |
| 47 | 339122 | RAB43 | 1083 | -0.462 | -0.3333 | Yes |
| 48 | 9939 | RBM8A | 1093 | -0.474 | -0.3312 | Yes |
| 49 | 11221 | DUSP10 | 1095 | -0.475 | -0.3229 | Yes |
| 50 | 3669 | ISG20 | 1097 | -0.475 | -0.3147 | Yes |
| 51 | 10098 | TSPAN5 | 1112 | -0.485 | -0.3161 | Yes |
| 52 | 2012 | EMP1 | 1119 | -0.494 | -0.3113 | Yes |
| 53 | 54541 | DDIT4 | 1133 | -0.502 | -0.3116 | Yes |
| 54 | 64795 | RMND5A | 1142 | -0.508 | -0.3080 | Yes |
| 55 | 7074 | TIAM1 | 1143 | -0.508 | -0.2984 | Yes |
| 56 | 639 | PRDM1 | 1159 | -0.523 | -0.2999 | Yes |
| 57 | 4084 | MXD1 | 1167 | -0.535 | -0.2950 | Yes |
| 58 | 2119 | ETV5 | 1176 | -0.545 | -0.2907 | Yes |
| 59 | 6310 | ATXN1 | 1177 | -0.547 | -0.2804 | Yes |
| 60 | 54407 | SLC38A2 | 1182 | -0.553 | -0.2729 | Yes |
| 61 | 56180 | MOSPD1 | 1186 | -0.558 | -0.2646 | Yes |
| 62 | 153222 | CREBRF | 1187 | -0.559 | -0.2541 | Yes |
| 63 | 57649 | PHF12 | 1212 | -0.588 | -0.2611 | Yes |
| 64 | 10150 | MBNL2 | 1217 | -0.591 | -0.2529 | Yes |
| 65 | 153 | ADRB1 | 1221 | -0.595 | -0.2439 | Yes |
| 66 | 10221 | TRIB1 | 1229 | -0.604 | -0.2378 | Yes |
| 67 | 8408 | ULK1 | 1243 | -0.622 | -0.2358 | Yes |
| 68 | 2355 | FOSL2 | 1262 | -0.648 | -0.2372 | Yes |
| 69 | 1358 | CPA2 | 1263 | -0.648 | -0.2249 | Yes |
| 70 | 8651 | SOCS1 | 1272 | -0.661 | -0.2184 | Yes |
| 71 | 7994 | KAT6A | 1279 | -0.666 | -0.2103 | Yes |
| 72 | 83931 | STK40 | 1281 | -0.669 | -0.1984 | Yes |
| 73 | 54800 | KLHL24 | 1287 | -0.677 | -0.1894 | Yes |
| 74 | 861 | RUNX1 | 1293 | -0.687 | -0.1802 | Yes |
| 75 | 55691 | FRMD4A | 1295 | -0.689 | -0.1679 | Yes |
| 76 | 3084 | NRG1 | 1320 | -0.739 | -0.1720 | Yes |
| 77 | 113791 | PIK3IP1 | 1345 | -0.787 | -0.1753 | Yes |
| 78 | 54463 | FAM134B | 1356 | -0.815 | -0.1674 | Yes |
| 79 | 54790 | TET2 | 1364 | -0.842 | -0.1568 | Yes |
| 80 | 4734 | NEDD4 | 1369 | -0.856 | -0.1436 | Yes |
| 81 | 9990 | SLC12A6 | 1379 | -0.922 | -0.1329 | Yes |
| 82 | 10397 | NDRG1 | 1380 | -0.925 | -0.1155 | Yes |
| 83 | 4919 | ROR1 | 1385 | -0.954 | -0.1004 | Yes |
| 84 | 1491 | CTH | 1391 | -1.004 | -0.0852 | Yes |
| 85 | 26959 | HBP1 | 1395 | -1.073 | -0.0672 | Yes |
| 86 | 2591 | GALNT3 | 1403 | -1.195 | -0.0498 | Yes |
| 87 | 51274 | KLF3 | 1406 | -1.296 | -0.0268 | Yes |
| 88 | 255394 | TCP11L2 | 1408 | -1.538 | 0.0015 | Yes |
Table: GSEA details [plain text format]

  

Fig 2: RTAAACA\_FREAC2\_01      
 Blue-Pink O' Gram in the Space of the Analyzed GeneSet

  

Fig 3: RTAAACA\_FREAC2\_01: Random ES distribution      
 Gene set null distribution of ES for **RTAAACA\_FREAC2\_01**
